# Education level and risk of postpartum depression: results from the Japan Environment and Children’s Study (JECS)

**DOI:** 10.1186/s12888-019-2401-3

**Published:** 2019-12-27

**Authors:** Kenta Matsumura, Kei Hamazaki, Akiko Tsuchida, Haruka Kasamatsu, Hidekuni Inadera, the Japan Environment and Children’s Study (JECS) Group, Michihiro Kamijima, Shin Yamazaki, Yukihiro Ohya, Reiko Kishi, Nobuo Yaegashi, Koichi Hashimoto, Chisato Mori, Shuichi Ito, Zentaro Yamagata, Hidekuni Inadera, Michihiro Kamijima, Takeo Nakayama, Hiroyasu Iso, Masayuki Shima, Youichi Kurozawa, Narufumi Suganuma, Koichi Kusuhara, Takahiko Katoh

**Affiliations:** 1 Toyama Regional Center for Japan Environment and Children’s Study, Faculty of Medicine, University of Toyama, 2630 Sugitani, Toyama, Toyama 930-0194 Japan; 2 Department of Public Health, Faculty of Medicine, University of Toyama, Toyama, Japan

**Keywords:** Perinatal depression, Postpartum anxiety, Longitudinal study, Income, Occupation

## Abstract

**Background:**

Lower socioeconomic status is often thought to be associated with an elevated risk of postpartum depression; however, this relationship exhibits noticeable heterogeneity between studies. The present study examined this relationship in Japan.

**Methods:**

Data were obtained from 90,194 mothers in an ongoing birth cohort of the Japan Environment and Children’s Study. Socioeconomic status was assessed based on the mothers’ highest education level during pregnancy. Postpartum depression was identified at 1 and 6 months postpartum based on an Edinburgh Postnatal Depression Scale score of ≥9, and analyses were also performed based on the sub-scores for anxiety, depression, and anhedonia symptoms. Logistic and generalized linear regression model analyses were used to calculate odds ratios for postpartum depression according to education level with the highest education group (≥16 years of education) defined as the reference group, while controlling for covariates in a stepwise fashion.

**Results:**

Univariate analysis revealed that a lower education level was associated with a higher prevalence of postpartum depression and related symptoms. Although these relationships weakened in the fully adjusted models, odds ratios for cases and related symptoms remained significant at 1 and 6 months postpartum. Among three symptom dimensions, the relationship was strongest and weakest in the depressive and anxiety symptoms, respectively.

**Conclusions:**

A lower education level was an independent risk factor for postpartum depression. In view of the low mobility of the education level, this finding suggests the potential importance of collecting information regarding education levels at the earliest opportunity.

## Background

The education level of an individual is one of the most frequently used indices for socioeconomic status [1], with lower socioeconomic status being related to increased risks of psychiatric diseases including depression [1–3], schizophrenia [3], anxiety disorders [4], and post-traumatic stress disorder [5]. However, this relationship is not always constant, and some studies have revealed that a lower education level is not related to a higher prevalence of major depression [6, 7]. This discrepancy is likely to be related to the influences of study time and location, as psychiatric problems are subject to cultural and biological factors [8, 9].

Postpartum depression, a major comorbidity among perinatal suicide victims [10, 11], also exhibits variability in its risk and protective factors. Some studies have also revealed that a lower education level is a risk factor for postpartum depression [12–14]; however, other studies did not detect this relationship [15, 16]. In view of the difficulty of modifying education levels, caregivers and researchers should understand whether it serves as a risk factor, neutral factor, or even protective factor for postpartum depression. However, the education level has rarely been evaluated as a main variable of interest [17], and further studies are needed to consider it in that context, rather than as a potential confounding factor.

When assessing postpartum depression, it is desirable to consider its complexity and diversity in terms of symptoms and peak periods. For instance, the Edinburgh Postnatal Depression Scale (EPDS) [18], which is widely used to screen for postpartum depression, has a three-factor structure that considers anxiety, depression, and anhedonia [19–22]. In addition, the prevalence of postpartum depression varies according to the interval from childbirth, with a peak at approximately 2–4 weeks postpartum [23–25]. However, a recent report has indicated that few studies have considered both aspects simultaneously [20].

Therefore, the present study evaluated the relationship between socioeconomic status, which was evaluated based on highest education level, and the prevalence of postpartum depression, as well as its symptoms and severity over time. The study data were obtained from a birth cohort of > 90,000 mothers from the Japan Environment and Children’s Study (JECS), which allowed the stable analyses to be controlled for many related factors and possible confounders.

## Methods

### Study design and participants

The detailed design and baseline characteristics of the JECS cohort have been published previously [26, 27]. The JECS is a nationwide government-funded birth cohort study that focuses on various environmental factors and child health and development. In the JECS, 103,062 pregnancies were registered via recruitment at 15 regional centers in both, rural and urban locations throughout Japan. The sample size was determined in advance to maintain adequate statistical power for evaluating conditions with a prevalence of ≤1%. The eligibility criteria for the pregnant women were as follows: 1) they resided in the study areas at recruitment and were expected to reside continually in Japan for the foreseeable future, 2) the expected delivery date was approximately between August 1, 2011 and mid-2014, and 3) they were capable of comprehending and completing the self-administered questionnaire. Women were excluded if they resided outside the study areas, even if they visited cooperating healthcare providers within the study areas. The study protocol was approved by the Ministry of the Environment’s Institutional Review Board on Epidemiological Studies and by the ethics committees of all participating institutions. All women provided written informed consent prior to participation.

The recruitment was performed between January 2011 and March 2014. Follow-up evaluation was primarily conducted at 1 month postpartum via mailed letters and scheduled in-hospital check-ups, and at 6 months postpartum via mailed letters. Data were acquired using self-administered questionnaires or medical record transcriptions performed by physicians, midwives/nurses, and/or research coordinators. The dataset that was used in the present study is named *jecs-an-20180131* (released in March 2018) and contains data from the first trimester, second/third trimester, 1-month follow-up, and 6-month follow-up.

**Fig. 1:**
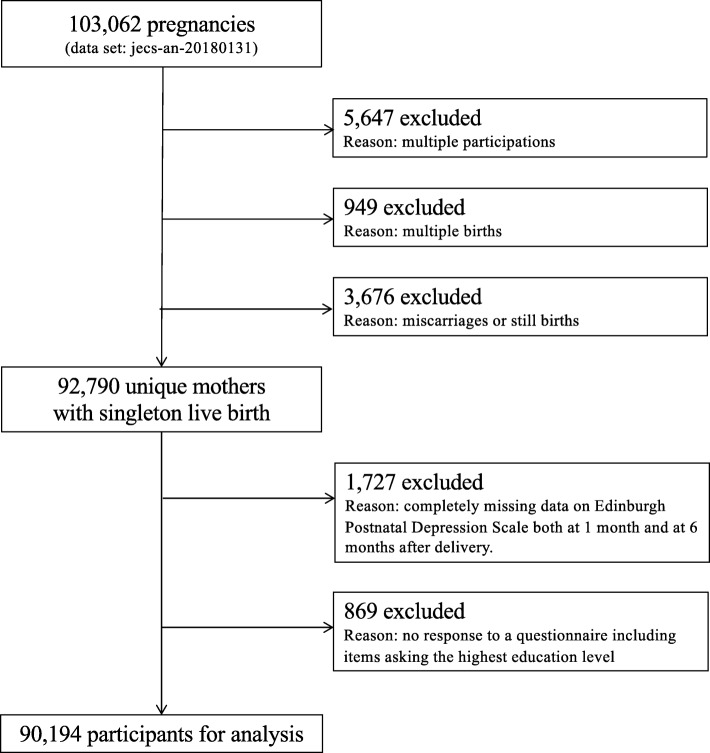
Study flow chart. See text for details

Among the 103,062 pregnancies in the dataset, 5647, 949, and 3676 were excluded owing to multiple registrations, multiple births, and miscarriage or stillbirth, respectively. Among the remaining 92,790 unique mothers with singleton live births, 1727 were excluded owing to completely missing data or no response to the 1- and 6-month EPDS questionnaires; 869 mothers were excluded owing to missing data regarding the highest education level during pregnancy. Therefore, the present study analyzed data from 90,194 unique mothers with singleton live births (Fig. 1).

### Measures

#### Exposure

Socioeconomic status was evaluated based on the women’s highest education level, as this factor is a more stable proxy for socioeconomic status than occupation or income, which frequently change during childbearing years [28]. The highest education level was categorized as ≥16 years (bachelor’s degree or postgraduate degree), >12–<16 years (technical junior college, technical/vocational college, or associate degree), or ≤12 years (junior high school or high school) of education. The data were collected during the second/third trimesters.

### Outcomes

Postpartum depression and its symptoms were assessed using the EPDS [18] at 1 and 6 months postpartum. The EPDS is a 10-item self-administered questionnaire that is used to screen for postpartum depression, with the score of each item and the total scores ranging from 0 to 3 (four-point scale) and 0 to 30, respectively. This tool is widely used, and has been translated into > 50 languages; the Japanese version developed by Okano et al. [29] using a back-translation technique provides good internal consistency (Cronbach’s alpha = 0.78) [30], test-retest reliability (*r* = 0.92), and an optimal cut-off score of 8/9 (75% sensitivity and 93% specificity). The present study also used the 8/9 cut-off point, which was validated in the study by Yamashita et al. [31] (82% sensitivity and 95% specificity) and has since been widely used to identify postpartum depression in Japan [20, 23–25, 32, 33].

Previous studies have evaluated the factor structure of EPDS [21, 34, 35], and the Japanese version of EPDS also likely has a three-factor structure that includes anxiety, depression, and anhedonia [19, 20]; however, there is some ambiguity regarding this relationship. Therefore, we conducted factor analysis using the maximum likelihood method and promax rotation, setting the number of predetermined factors to 3; this is consistent with the methods used in previous studies [19, 20]. We then defined the sum of the relevant items as “anxiety” (EPDS items 3 = self-blame, 4 = anxious, and 5 = scared), “depressive symptoms” (items 7 = hard to sleep, 9 = crying, and 10 = self-harm), and “anhedonia” (items 1 = laugh and 2 = enjoyment), based on the subscale rule of items having a factor loading of ≥0.4 for a particular factor and < 0.3 for other factors. The results of the confirmatory factor analysis have been provided in (Additional file 1: Figure S1).

### Covariates

We selected both potential confounders, defined as variables impacting on both, exposure and outcome, and potential mediators, defined as variables mediating exposure and outcome, in this study. Firstly, we chose physician-diagnosed histories of depression (yes or no), anxiety disorder (yes or no), dysautonomia (yes or no), and schizophrenia (yes or no) as potential confounding covariates. These were known as risk factors for postpartum depression [36], as they could interrupt academic learning. Secondly, we chose the following variables for potential mediating covariates: maternal age (continuous years), body mass index (<18.5, 18.5–<25, and ≥25 kg/m^2^), smoking status (never, former, and current), alcohol intake (never, former, current at the rate of 1–3 times/month, and current at the rate of ≥1 times/week), physical activity (continuous METs × h/day), employment status (yes or no), parity (primipara or multipara), marital status (married, single, and divorced or widowed), passive smoking status (never, pre-pregnancy, and during pregnancy), annual household income (<4, 4–<6, ≥6 million Japanese yen), and feeding method at 1 month (exclusive breastfeeding, mixed feeding, or only formula feeding). These covariates could be affected by the education level, and were regarded as risk factors for postpartum depression [36].

The variables were categorized according to standard medical practice, common practice in Japan, and/or based on previous studies [32, 37, 38].

### Statistical analysis

The outcome variables at 1 and 6 months postpartum were cases of postpartum depression (defined as any woman with an EPDS of ≥9), the total EPDS score (summated scores for items 1–10), and the sub-scores for anxiety (EPDS items 3, 4, and 5), depressive symptoms (items 7, 9, and 10), and anhedonia (items 1 and 2). As mentioned previously, the exposure variable was defined as the mother’s highest education level (≥16 years, >12–<16 years, and ≤12 years).

Logistic regression analysis was used to calculate the crude and adjusted odds ratios (CORs and AORs) and their corresponding 95% confidence intervals (CIs) for the cases. Generalized linear regression models, setting the logit as a link function after transforming each score into a ratio value (e.g., dividing the total score by 30 and the depression subscale by 9), were used to calculate the CORs and AORs and their 95% CIs for EPDS scores (i.e., total, anxiety, depression, and anhedonia). This analysis corresponds to an extension of logistic analysis when outcomes may be counted by numbers; that is, when the EPDS score distributes binomially rather than normally. In either analysis, the group with ≥16 years of education was considered as the reference group.

The forced entry method was used to include covariates in the multivariate analysis. In model 1, the regression models were adjusted only for the potential confounding covariates. Hence, the AOR from this model was referred to as AOR1. In model 2, the models were adjusted for the potential mediating covariates in addition to the covariates used in model 1. Hence, the AOR from this model was referred to as AOR2.

All analyses were performed using SAS software (version 9.4; SAS Institute Inc., Cary, NC).

### Missing data

The response rates were 99.57% at 1 month postpartum (*n* = 89,803) and 94.72% at 6 months postpartum (*n* = 85,431), with only 0.43% (*n* = 391) of the women responding to the 6-month follow-up, but not the 1-month follow-up. Among the 90,194 included pregnancies, the missing data rate was < 1% for most covariates, with the exceptions of physical activity (4.94%, *n* = 4457) and annual household income (7.17%, *n* = 6470). The missing data rate for the exposure measure (highest education level) was 0.57% (*n* = 517). Each of the 10 items from the Japanese EPDS had missing data rates of < 0.90% at 1 month (maximum *n* = 809); however, 1.95% of the cases (*n* = 1756) had at least one missing value. The EPDS items had average missing data rates of up to 5.70% at 6 months (maximum *n* = 5253); however, 6.66% (*n* = 6007) had at least one missing value. A total of 18,167 (20.14%) mothers had at least one missing value.

Data imputation was performed using chained equations [39] to create 10 imputed datasets, with the data imputed simultaneously irrespective of the measurement time points. When conducting multiple imputations, auxiliary variables that were related to the analyzed variables were also included to preserve the assumption of data missing at random.

### Sensitivity analysis

The patterns of the resulting ORs for the complete datasets (*n* = 76,716 at 1 month and *n* = 72,809 at 6 months) were compared to those from the multiply imputed datasets (both *n* = 90,194) to assess the differences between the strategies for addressing missing values.

## Results

A total of 90,194 mothers were analyzed in this study. Their mean age was 31.3 ± 5.04 (SD) years; the BMI before pregnancy was 21.21 ± 3.28 (SD), 43.7% of mothers were primipara, and 95.5% were married. Overall, 21.8% (*n* = 19,538), 42.2% (*n* = 37,832) and 36.0% (*n* = 32,307) mothers had ≥16, >12–<16, and ≤12 years’ education, respectively. Compared to those included (*n* = 90,194), mothers who were excluded from the analysis (*n* = 2596) tended to be younger (Cohen’s *d* = 0.22) and had a higher rate of current smokers (Cramer’s *V* = .06). The details of the participants’ characteristics according to the education level are presented in Table 1. The highest education level was associated with annual household income, smoking status, passive smoking, and employment status above the level of small effect size (Cramer’s *V* ≥ .10).

**Table 1:** Characteristics of participants according to education level

		Highest education level					
		≥16 years		>12–<16 years		≤12 years	
		*n* (%)		*n* (%)		*n* (%)	
Subtotal		19,538	(21.8)	37,832	(42.2)	32,307	(36.0)
Mothers							
Age, years	n	19,470		37,696		32,110	
	Mean	32.5		31.9		29.9	
	± SD	± 4.1		± 4.6		± 5.6	
BMI, kg/m^2^	<18.5	3340	(17.1)	5890	(15.6)	5278	(16.4)
	18.5–<25	14,952	(76.6)	28,132	(74.4)	22,640	(70.1)
	≥25	1236	(6.3)	3793	(10.0)	4362	(13.5)
Parity	Primipara	9694	(49.6)	16,502	(43.6)	12,958	(40.1)
	Multipara	9839	(50.4)	21,314	(56.4)	19,333	(59.9)
Smoking status	Never	15,253	(78.6)	23,354	(62.1)	13,432	(42.0)
	Former	4062	(20.9)	13,380	(35.6)	15,735	(49.3)
	Current	104	(0.5)	851	(2.3)	2784	(8.7)
Alcohol intake	Never	18,145	(93.4)	34,771	(92.4)	28,392	(88.7)
	Former	734	(3.8)	1565	(4.2)	1741	(5.4)
	Current (1–3 times / month)	417	(2.2)	905	(2.4)	1196	(3.7)
	Current (≥1 times / week)	135	(0.7)	379	(1.0)	680	(2.1)
Physical activity,	n	18,754		35,977		30,689	
METs h/day	Mean	2.8		4.4		4.2	
	± SD	± 5.5		± 9.0		± 8.8	
Employed	No	7584	(39.0)	15,980	(42.5)	17,166	(53.7)
	Yes	11,864	(61.0)	21,621	(57.5)	14,792	(46.3)
History of depression	No	18,892	(97.1)	36,700	(97.5)	30,965	(96.3)
	Yes	557	(2.9)	961	(2.6)	1181	(3.7)
History of anxiety disorder	No	19,022	(97.8)	36,731	(97.5)	30,975	(96.4)
	Yes	427	(2.2)	930	(2.5)	1171	(3.6)
History of dysautonomia	No	18,837	(96.9)	36,403	(96.7)	30,706	(95.5)
	Yes	612	(3.2)	1258	(3.3)	1440	(4.5)
History of schizophrenia	No	19,422	(99.9)	37,609	(99.9)	32,072	(99.8)
	Yes	27	(0.1)	52	(0.1)	74	(0.2)
Feeding method	Breastfeeding only	9009	(46.4)	16,402	(43.7)	12,047	(37.6)
	Mixed feeding	10,288	(53.0)	20,826	(55.4)	19,158	(59.8)
	Formula only	113	(0.6)	349	(0.9)	813	(2.5)
Family							
Marital Status	Married	19,065	(98.2)	36,430	(97.1)	29,419	(92.2)
	Single	320	(1.7)	966	(2.6)	1913	(6.0)
	Divorced or widowed	33	(0.2)	142	(0.4)	581	(1.8)
Annual household income,	<4	3941	(20.9)	13,030	(36.7)	16,460	(56.3)
million yen	4–<6	6231	(33.0)	12,829	(36.1)	8589	(29.4)
	≥6	8706	(46.1)	9640	(27.2)	4168	(14.3)
Passive smoking	No	12,818	(66.0)	18,722	(49.8)	10,793	(33.7)
	Outdoors	6467	(33.3)	18,171	(48.3)	19,894	(62.2)
	Yes	152	(0.8)	694	(1.9)	1323	(4.1)

All *p* values of the results of χ^2^ test and analysis of variance were < .001

*SD* standard deviation, *BMI* body mass index, *METs* metabolic equivalents

The prevalence of postpartum depression (defined as EPDS total score ≥ 9) at 1 and 6 months postpartum were 14.5 and 11.8%, respectively. Overall, logistic regression analysis revealed a tendency for the ORs to increase with a decrease in the education level (e.g., 1 month: AOR1 [CI] = 1.14 [1.08–1.20] for >12–< 16 years group, AOR1 [CI] = 1.48 [1.40–1.56] for ≤12 years group); however, it decreased according to the increase in the number of covariates adjusted (i.e., crude model to model 1 to model 2). The linear trend was significant at the level of *p* < .001 in all models. The prevalence, cases, and ORs for postpartum depression according to the education level at 1 and 6 months postpartum are summarized in Table 2.

**Table 2:** Prevalence, cases, and ORs for postpartum depression assessed using EPDS according to education level

	Highest education level						*p*-value for trend
	≥16 years (*n* = 19,621)		>12–<16 years (*n* = 38,030)		≤12 years (*n* = 32,543)		
1 month							
Prevalence, %	11.6		13.2		17.7		
Cases, n	2271		5010		5768		
OR (95% CI)							
Crude	1.00	–	**1.16**	**(1.10–1.22)**	**1.65**	**(1.56–1.73)**	**< .001**
Model 1	1.00	–	**1.14**	**(1.08–1.20)**	**1.48**	**(1.40–1.56)**	**< .001**
Model 2	1.00	–	**1.07**	**(1.01–1.13)**	**1.26**	**(1.19–1.34)**	**< .001**
6 months							
Prevalence, %	9.0		10.5		15.2		
Cases, n	1768		3975		4933		
OR (95% CI)							
Crude	1.00	–	**1.18**	**(1.11–1.25)**	**1.80**	**(1.70–1.92)**	**< .001**
Model 1	1.00	–	**1.15**	**(1.09–1.23)**	**1.57**	**(1.48–1.67)**	**< .001**
Model 2	1.00	–	1.03	(0.97–1.10)	**1.21**	**(1.13–1.29)**	**< .001**

The table shows the imputed data for the 90,194 mothers in the study

Boldface indicates statistical significance at the level of 5%

*OR* odds ratio, *CI* confidence interval, *EPDS* Edinburgh Postnatal Depression Scale

Cases: a total EPDS score of ≥9

Crude: crude model

Model 1: Partial model adjusted for physician-diagnosed history of depression, anxiety disorder, dysautonomia, and schizophrenia

Model 2: Full model adjusted for all the covariates of the model 1; maternal age; body mass index; parity; smoking status; alcohol intake; physical activity; employment status; feeding method; marital status; annual household income; and passive smoking status

The mean values of total EPDS, anxiety, depression, and anhedonia scores at 1 month postpartum were 5.14 ± 3.54, 2.94 ± 1.94, 0.35 ± 0.98, and 0.19 ± 0.60, respectively. Those at 6 months postpartum were 4.65 ± 3.54, 2.67 ± 1.93, 0.42 ± 1.10, and 0.09 ± 0.42, respectively. Similar to the results of the cases, generalized linear regression model analysis revealed an overall tendency of the ORs to increase with a decrease in the education level (e.g., EPDS depression at 1 month: AOR1 [CI] = 1.17 [1.13–1.22] for >12–< 16 years group, AOR1 [CI] = 1.75 [1.69–1.81] for ≤12 years group); however, it decreased with an increase in the number of covariates adjusted. The prevalence, cases, total score, and symptoms of postpartum depression and their CORs and AORs according to education level at 1 month and 6 months postpartum, are summarized in Table 3.

**Table 3:** Mean (SD) scores and ORs for the symptoms of postpartum depression according to education level

	Highest education level						*p*-value for trend
	≥16 years		>12–<16 years		≤12 years		
	(*n* = 19,621)		(*n* = 38,030)		(*n* = 32,543)		
1 month							
EPDS total							
Mean (SD)	4.83 (3.26)		5.01 (3.39)		5.49 (3.86)		
OR (95% CI)							
Crude	1.00	––––	**1.04**	**(1.04–1.05)**	**1.17**	**(1.16–1.18)**	**< .001**
Model1	1.00	––––	**1.04**	**(1.03–1.05)**	**1.12**	**(1.11–1.13)**	**< .001**
Model2	1.00	––––	**1.01**	**(1.00–1.02)**	**1.05**	**(1.04–1.07)**	**< .001**
EPDS anxiety							
Mean (SD)	2.79 (1.87)		2.88 (1.91)		3.08 (2.02)		
OR (95% CI)							
Crude	1.00	––––	**1.05**	**(1.04–1.06)**	**1.16**	**(1.15–1.18)**	**< .001**
Model1	1.00	––––	**1.04**	**(1.03–1.05)**	**1.10**	**(1.08–1.11)**	**< .001**
Model2	1.00	––––	**1.02**	**(1.01–1.03)**	**1.05**	**(1.04–1.07)**	**< .001**
EPDS depression							
Mean (SD)	0.25 (0.80)		0.29 (0.88)		0.47 (1.16)		
OR (95% CI)							
Crude	1.00	––––	**1.20**	**(1.16–1.24)**	**1.97**	**(1.91–2.04)**	**< .001**
Model1	1.00	––––	**1.17**	**(1.13–1.22)**	**1.75**	**(1.69–1.81)**	**< .001**
Model2	1.00	––––	**1.04**	**(1.01–1.08)**	**1.31**	**(1.26–1.36)**	**< .001**
EPDS anhedonia							
Mean (SD)	0.18 (0.56)		0.19 (0.59)		0.22 (0.64)		
OR (95% CI)							
Crude	1.00	––––	**1.06**	**(1.02–1.11)**	**1.24**	**(1.19–1.29)**	**< .001**
Model1	1.00	––––	**1.07**	**(1.02–1.11)**	**1.24**	**(1.19–1.30)**	**< .001**
Model2	1.00	––––	1.03	(0.98–1.07)	**1.11**	**(1.06–1.16)**	**< .001**
6 months							
EPDS total							
Mean (SD)	4.35 (3.19)		4.50 (3.32)		5.01 (3.97)		
OR (95% CI)							
Crude	1.00	––––	**1.04**	**(1.03–1.05)**	**1.18**	**(1.17–1.19)**	**< .001**
Model1	1.00	––––	**1.03**	**(1.02–1.04)**	**1.12**	**(1.11–1.14)**	**< .001**
Model2	1.00	––––	**1.00**	**(0.99–1.00)**	**1.03**	**(1.02–1.04)**	**< .001**
EPDS anxiety							
Mean (SD)	2.53 (1.82)		2.61 (1.88)		2.82 (2.05)		
OR (95% CI)							
Crude	1.00	––––	**1.04**	**(1.03–1.05)**	**1.16**	**(1.15–1.18)**	**< .001**
Model1	1.00	––––	**1.03**	**(1.02–1.05)**	**1.10**	**(1.09–1.12)**	**< .001**
Model2	1.00	––––	1.00	(0.98–1.01)	**1.02**	**(1.01–1.04)**	**< .001**
EPDS depression							
Mean (SD)	0.32 (0.92)		0.36 (0.99)		0.56 (1.34)		
OR (95% CI)							
Crude	1.00	––––	**1.16**	**(1.12–1.19)**	**1.82**	**(1.76–1.88)**	**< .001**
Model1	1.00	––––	**1.13**	**(1.09–1.17)**	**1.57**	**(1.52–1.63)**	**< .001**
Model2	1.00	––––	1.00	(0.97–1.04)	**1.18**	**(1.14–1.23)**	**< .001**
EPDS anhedonia							
Mean (SD)	0.08 (0.38)		0.08 (0.40)		0.11 (0.50)		
OR (95% CI)							
Crude	1.00	––––	**1.13**	**(1.06–1.20)**	**1.49**	**(1.39–1.59)**	**< .001**
Model1	1.00	––––	**1.13**	**(1.06–1.21)**	**1.47**	**(1.37–1.57)**	**< .001**
Model2	1.00	––––	1.02	(0.96–1.09)	**1.14**	**(1.06–1.23)**	**< .001**

The table shows the imputed data for the 90,194 mothers in the study

Boldface indicates statistical significance at the level of 5%

OR odds ratio, CI confidence interval, EPDS Edinburgh Postnatal Depression Scale

Crude: crude model

Model 1: Partial model adjusted for physician-diagnosed history of depression, anxiety disorder, dysautonomia, and schizophrenia

Model 2: Full model adjusted for all the covariates of the model 1: maternal age; body mass index; parity; smoking status; alcohol intake; physical activity; employment status; feeding method; marital status; annual household income; and passive smoking status

It is important to note that the prevalence of postpartum depression was evaluated on a binary scale, whereas the scores and symptoms were evaluated on interval scales; this may have influenced the magnitudes of the various ORs.

The results of the sensitivity analysis using the complete case dataset were not meaningfully different from those calculated using the multiply imputed dataset. These results are available in (Additional file 2: Tables S1) and (Additional file 3: Table S2).

## Discussion

The present study examined the association between the highest education level, which could be regarded as a stable proxy for socioeconomic status for mothers at childbearing age, and the prevalence of postpartum depression and its subcategory symptoms at 2 time points using nationwide data from a JECS birth cohort; up to 15 covariates were controlled during the analysis [26, 27]. Univariate analyses (crude model) revealed that a lower education level was associated with a higher prevalence of postpartum depression and related symptoms of anxiety, depression, and anhedonia. Although these relationships weakened with an increase in the number of covariates entered (i.e., crude model to partially adjusted model to fully adjusted model), the ORs for cases and all three symptoms remained significant in the fully adjusted model at both, 1 and 6 months postpartum. These findings suggest that lower socioeconomic status is an independent risk factor for postpartum depression.

In view of the low mobility of the education level, it is difficult to raise education levels during pregnancy. However, in view of the present results, the education level may be used for screening mothers who are at a risk of postpartum depression. Fortunately, it is easier to obtain information regarding the education level than other socioeconomic status-related variables, such as income; the missing value rate of income (7.17%) was approximately 12-fold higher than that of the education level (0.57%). Therefore, it is recommended that in addition to the usual variables, caregivers should collect information pertaining to the education level at the earliest opportunity.

A lower education level was associated with more symptoms of severe depression (e.g., AOR1 [CI] = 1.75 [1.69–1.81] for ≤12 years group at 1 month); however, it was not as strongly associated with anxiety (e.g., AOR1 [CI] = 1.10 [1.08–1.11] for ≤12 years group at 1 month), and anhedonia (e.g., AOR1 [CI] = 1.24 [1.19–1.30] for ≤12 years group at 1 month). To the best of our knowledge, our study is the first to evaluate the relationships between the education level and multiple symptoms of postpartum depression. Since the crude ORs for anxiety and anhedonia were closer to 1 than that for the symptoms of depression, the relationships between the education level and anxiety and anhedonia were relatively weak. It is unclear as to why this discrepancy exists; however, some researchers argue that there is a difference between the pathogenesis of depression itself in postpartum depression and the so-called “postpartum anxiety,” that is currently grouped with postpartum depression [40–42]. Further studies are needed to examine whether this interesting difference is temporarily stable and/or observed in other countries; this could provide information regarding the pathology of postpartum depression.

In this study, the histories of depression and anxiety were all approximately 2–3%; this appears considerably low. However, according to a cross-national study [6], Japan showed the lowest prevalence rate of episodes of DSM-IV major depression among 18 countries, including both high-income and low- to middle-income countries. Thus, the present 2–3% rate of depression is not likely to be exceptionally low. In addition, Japan also showed a lower prevalence of anxiety [43, 44]. Similarly, the present 2–3% rate of anxiety is not likely to be exceptionally low.

The present study has several strengths. First, it had a large sample size (*N* ≥ 90,000 patients). To the best of our knowledge, this is the largest birth cohort study to examine the relationship between the education level and postpartum depression in expectant mothers. Second, the study was conducted nationwide. The participants were recruited via 15 regional centers in both, rural and urban locations throughout Japan. Therefore, the sample may be considered to be highly representative of the Japanese population of expectant mothers. Finally, in the present study, postpartum depression was assessed in terms of the symptoms of anxiety, depression, and anhedonia at 1 and 6 months postpartum. Till date, few studies have considered both aspects, i.e., symptoms and timepoints, simultaneously.

The present study also has several limitations. First, we evaluated postpartum depression using self-administered EPDS questionnaires; it is possible that the prevalence of postpartum depression would have differed if it were based on clinical diagnoses. A previous review revealed a high prevalence using the Beck Depression Inventory for assessing postpartum depression; however, that tendency was not obvious when using the EPDS [45]. Second, by excluding women on the lower end of the education spectrum (who could not complete the self-administered questionnaires), we probably excluded the group that is most susceptible to poor outcomes; this would have attenuated the observed relationships. Third, smokers were a minority overall; however, such mothers were likely to be excluded from the analysis. Therefore, the present findings may not hold true for smokers. Fourth, the assessments were performed at 1 and 6 months postpartum; the 6-month assessment is technically beyond the standard postpartum depression period [46]. However, experts often assert that this period should be extended to 12 months [47]. Finally, since the present study was not a randomized control trial, the observed relationships should not be considered to be directly causative; however, potential confounding covariates were controlled in the adjusted models.

## Conclusions

We found that a lower education level was univariately associated with a higher prevalence of postpartum depression and symptoms of anxiety, depression, and anhedonia at both, 1 and 6 months postpartum. Although these relationships weakened on multivariate analyses as the number of covariates increased, they remained significant to the end. Interestingly, the relationship was strongest for symptoms of depression and weakest for symptoms of anxiety. These findings suggest that lower socioeconomic status is an independent risk factor for postpartum depression. In view of the difficulty of directly modifying the education level, in addition to the usual variables, caregivers should collect information regarding the education level at the earliest opportunity.

### Supplementary information

**Additional file 1: Figure S1.** Confirmatory factor analysis of the Edinburgh Postnatal Depression Scale (EPDS), with standardized parameter estimates.

**Additional file 2: Table S1.** Prevalence, cases, and ORs for postpartum depression assessed using EPDS according to education level (complete case analysis).

**Additional file 3: Table S2.** Mean (SD) scores and ORs for the symptoms of postpartum depression according to education level (complete case analysis).

## Data Availability

The data used to derive our conclusions are unsuitable for public deposition because of ethical restrictions and the specific legal framework in Japan. Furthermore, the Ethical Guidelines for Epidemiological Research enforced by the Japanese Ministry of Education, Culture, Sports, Science, and Technology and the Ministry of Health, Labour, and Welfare restrict the open sharing of epidemiological data. All inquiries about access to data should be sent to: jecs-en@nies.go.jp. The person responsible for handling enquiries sent to this e-mail address is Dr. Shoji F. Nakayama, JECS Programme Office, National Institute for Environmental Studies.

## Appendix

Members of the JECS as of 2019: Michihiro Kamijima (principal investigator, Nagoya City University, Nagoya, Japan), Shin Yamazaki (National Institute for Environmental Studies, Tsukuba, Japan), Yukihiro Ohya (National Center for Child Health and Development, Tokyo, Japan), Reiko Kishi (Hokkaido University, Sapporo, Japan), Nobuo Yaegashi (Tohoku University, Sendai, Japan), Koichi Hashimoto (Fukushima Medical University, Fukushima, Japan), Chisato Mori (Chiba University, Chiba, Japan), Shuichi Ito (Yokohama City University, Yokohama, Japan), Zentaro Yamagata (University of Yamanashi, Chuo, Japan), Hidekuni Inadera (University of Toyama, Toyama, Japan), Michihiro Kamijima (Nagoya City University, Nagoya, Japan), Takeo Nakayama (Kyoto University, Kyoto, Japan), Hiroyasu Iso (Osaka University, Suita, Japan), Masayuki Shima (Hyogo College of Medicine, Nishinomiya, Japan), Youichi Kurozawa (Tottori University, Yonago, Japan), Narufumi Suganuma (Kochi University, Nankoku, Japan), Koichi Kusuhara (University of Occupational and Environmental Health, Kitakyushu, Japan), and Takahiko Katoh (Kumamoto University, Kumamoto, Japan).

## Abbreviations

AOR: Adjusted OR
AOR1: AOR for model 1
AOR2: AOR for model 2
BMI: Body mass index
CI: Confidence interval
COR: Crude OR
EPDS: Edinburgh Postnatal Depression Scale
JECS: Japan Environment and Children’s Study
METs: Metabolic equivalents
OR: Odds ratio
SD: Standard deviation
