# Supplementary material for: Education level and risk of postpartum depression: results from the Japan Environment and Children’s Study (JECS)
Source: BMC Psychiatry. 2019 Dec 27;19:419. doi: 10.1186/s12888-019-2401-3 (PMC6935197; doi:10.1186/s12888-019-2401-3)
Supplement: Supplementary file 1 — Additional file 1: Figure S1. Confirmatory factor analysis of the Edinburgh Postnatal Depression Scale (EPDS), with standardized parameter estimates. [file 12888_2019_2401_MOESM1_ESM.docx]

**Fig. S1** Confirmatory factor analysis of the Edinburgh Postnatal Depression Scale (EPDS), with standardized parameter estimates. Above and below values or numerator/denominator represent 1 month and 6 months postpartum, respectively.

AGFI: adjusted goodness-of-fit index; RMSEA: root mean square error of estimation; CFI: comparative fit index, TLI: Tucker-Lewis index, also known as the non-normed fit index (NNFI), SRMR: standardized root mean square residual.
