# Supplementary material for: Education level and risk of postpartum depression: results from the Japan Environment and Children’s Study (JECS)
Source: BMC Psychiatry. 2019 Dec 27;19:419. doi: 10.1186/s12888-019-2401-3 (PMC6935197; doi:10.1186/s12888-019-2401-3)
Supplement: Supplementary file 2 — Additional file 2: Table S1. Prevalence, cases, and ORs for postpartum depression assessed using EPDS according to education level (complete case analysis). [file 12888_2019_2401_MOESM2_ESM.docx]

**Table S1** Prevalence, cases, and ORs for postpartum depression assessed using EPDS according to education level (complete case analysis)

Boldface indicates statistical significance at the level of 5%.

OR: odds ratio, CI: confidence interval, EPDS: Edinburgh Postnatal Depression Scale.

Cases: a total EPDS score of ≥9.

Crude: crude model.

Model 1: Partial model adjusted for physician-diagnosed history of depression, anxiety disorder, dysautonomia, and schizophrenia.

Model 2: Full model adjusted for all the covariates of the model 1; maternal age; body mass index; parity; smoking status; alcohol intake; physical activity; employment status; feeding method; marital status; annual household income; and passive smoking status.
